# The Effect of Different Topical Agents (Silver Sulfadiazine, Povidone-Iodine, and Sodium Chloride 0.9%) on Burn Injuries in Rats

**DOI:** 10.1155/2014/907082

**Published:** 2014-09-29

**Authors:** Emir Burak Yüksel, Alpagan Mustafa Yıldırım, Ali Bal, Tuncay Kuloglu

**Affiliations:** ^1^Department of Plastic, Reconstructive & Esthetic Surgery, Elbistan State Hospital, Kahramanmaras, Turkey; ^2^Department of Plastic, Reconstructive & Esthetic Surgery, Afyon Kocatepe University, Afyon, Turkey; ^3^Department of Plastic, Reconstructive & Esthetic Surgery, Malatya State Hospital, Malatya, Turkey; ^4^Department of Histology & Embryology, Firat University, Elazıg, Turkey

## Abstract

It was aimed to comparatively evaluate the effects of dressing methods with silver sulfadiazine, povidone-iodine, and saline which have a common use in routine practices for burn injuries. Twenty-eight Sprague Dawley adult female rats were used in this study. All the rats were divided into 4 groups: the control group, the povidone-iodine group, the saline group, and the silver sulfadiazine group. On each rat, a second degree burn which covered less than 10% of the body surface area was created under general anesthesia by a metal comb including four probes with 2 × 1 cm area. The control group did not have any treatment during the experiment. Povidone-iodine, saline, and silver sulfadiazine administrations were performed under ether anesthesia every day. On 0, 7th, 14th, and 21st days of the study, tissue samples were taken for histological analyses. The sections taken from the paraffin blocks were stained and avidin-biotin-peroxidase method was used for collagen immune-reactivity. In the light microscope analyses, number of inflammatory cells, vascularization, fibroblast proliferation, collagen formation and epithelialization were evaluated histologically in all groups and analysed statistically. The agents that we used for injury healing in the treatment groups did not show any significant better results in comparison with the control group. In conclusion, further studies with the use of sodium chloride, silver sulfadiazine, and povidone-iodine by creating deeper and/or larger burn injury models are needed in order to accept these agents in routine treatment.

## 1. Introduction

Many types of medications have been used for burn injuries so far [1]. The common characteristics of these medications are that they all have antimicrobial effects. There are many studies conducted on the effects of these medications, which demonstrates that they insert their effects through various mechanisms. In this study, the effects of the most frequently used medications, that is, the silver sulfadiazine cream, antiseptic solution povidone-iodine, and 0.9% NaCl serum physiologic on the process of healing of the burn injuries, have been compared and examined [2–4].

## 2. Material and Method

Twenty-eight female adult Sprague-Dawley rats obtained from Firat University Experimental Researches Center (FÜDAM) have been used in the study.

**Figure 1:**
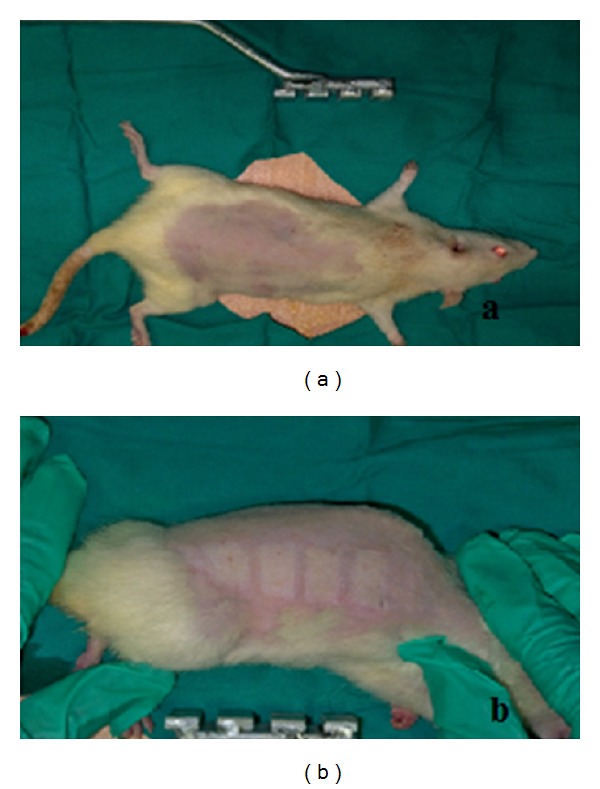
(a) The shaved view of the rats before the burn injury (b) and the view after the burn injury.

**Figure 2:**
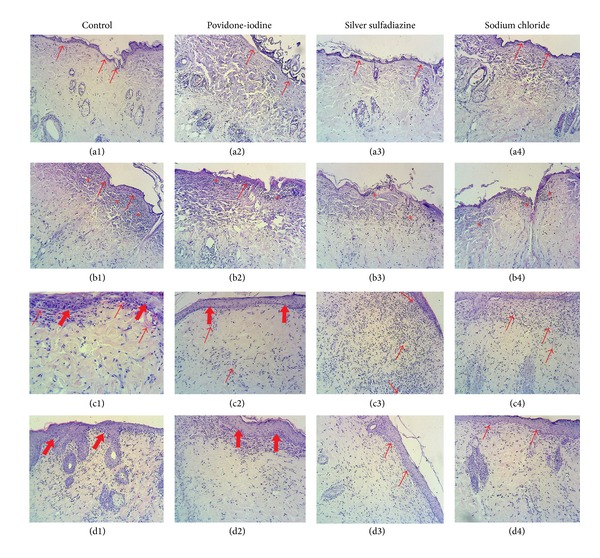
Day 0 ((a1), (a2), (a3), and (a4)), day 7 ((b1), (b2), (b3), and (b4)), day 14 ((c1), (c2), (c3), and (c4)), and day 21 ((d1), (d2), (d3), and (d4)) of hematoxylin and eosin staining. The arrows in (a1), (a2), (a3), and (a4) show the epidermis damage. The arrows in (b1), (b2), (b3), and (b4) show slight epithelisation, the star (∗) shows inflammatory cell infiltration. The thin arrows in (c1), (c2), (c3), and (c4) (→) show the vascularization; the thick arrows show the epithelisation. The thick arrows in (d1), (d2), (d3), and (d4) show the epithelisation. (×100).

The rats were separated into 4 groups each one consisting of 7 rats: the control group, 10% povidone-iodine group, 0.9% sodium chloride (Sf) group, and 1% silver sulfadiazine group. In all groups, second degree burns were induced on the shaved backs of the rats by pressing 4 metal plates (2 × 1 cm) after being kept in boiling water for 30 seconds. The metal plates were kept for 10 seconds on the backs of the rats, and the burns did not exceed 10% of the body surfaces (Figures 1 and 2). 2 mg/kg paracetamol was added to their drinking water as analgesic. In the control group, the burn injury was covered with sterile gauze bandage in day 0, after the burn injury was performed. No treatments were applied during the experiment. Only the medical dressing was changed during the days of biopsy.

In the “10% povidone-iodine” group, 10% povidone-iodine was applied to the burn injury every day under ether aesthesia. The injury area was covered with sterile gauze bandage and this process continued for 21 days.

In the “1% silver sulfadiazine” group, silver sulfadiazine was applied to the burn injury every day under ether aesthesia. The injury area was covered with sterile gauze bandage and this process continued for 21 days.

In the “0.9% sodium chloride” group, the injury area was moisturized with serum physiologic twice everyday under ether aesthesia, and the injury area was covered with sterile gauze bandage, and this process continued for 21 days.

On the 0, 7th, and 21st day of the experiment, tissue samples were taken under anesthesia from predetermined areas from all subjects in all groups.

Histological study: the tissue samples taken from each group were stained with Hematoxylin-eosin (H&E) and Masson trichrome and assessed in light microscopy. For the assessment of the recovery with immunohistochemical study, the collagen I immune-reactivity was performed using the avidin-biotin-peroxidase complex method.

### 2.1. Statistical Analysis

The histological assessment results were analysed with one sample Kolmogorov-Smirnov test. Since the groups showed normal distribution, the parametric statistics methods were used for the analysis of the data. The one-way ANOVA test was applied, and the Bonferroni test was used for the post hoc comparisons. The value *P* < 0.05 was accepted as statistically significant. The SPSS 12.0 statistical package program was used for the analysis of the data.

## 3. Findings

In the light microscopy examinations of control group, it was observed on day 0 that there were no significant changes in fibroblast proliferation, collagen formation, vascularization, epithelisation, and inflammatory cell density. On the other hand, it was observed that the epidermis layer was damaged due to the burn injury (Figures 2(a1), 3(a1), and 4(a1)). On the 7th day of the control group, severe inflammatory cell increase was observed, and in some subjects a slight increase in fibroblast proliferation, vascularization, and epithelisation was observed (Figures 2(b1), 3(b1), and 4(b1)). On the 14th day of the control group, a decrease was observed in the inflammatory cell density; and the fibroblast proliferation, vascularization, and collagen formation were obvious. Moreover, the epithelisation level was detected at medium level (Figures 2(c1), 3(c1), and 4(c1)). On the 21st day of control group, a decrease in the vascularization and an increase in inflammatory cell number were determined and the epithelisation, fibroblast proliferation, and collagen formation were observed (Figures 2(d1), 3(d1), and 4(d1)).

On day 0 of the 10% povidone-iodine group no changes were observed in fibroblast proliferation, collagen formation, vascularization, epithelisation, and inflammatory cell density; and the epidermis layer was observed to be severely damaged (Figures 2(a2), 3(a2), and 4(a2)). On the 7th day of the 10% povidone-iodine group, severe inflammatory cell infiltration was observed, and in some subjects, a slight increase was observed in fibroblast proliferation, vascularization, and epithelisation (Figures 2(b2), 3(b2), and 4(b2)). On the 14th day of the 10% povidone-iodine group, a decrease was observed in the inflammatory cell infiltration, and the fibroblast proliferation, vascularization, and collagen formation were obvious. The epithelisation was detected at medium level (Figures 2(c2), 3(c2), and 4(c2)). On the 21st day of the 10% povidone-iodine group, a decrease was observed in vascularization and inflammatory cell infiltration, and the epithelisation, fibroblast proliferation, and collagen formation were observed as severe (Figures 2(d2), 3(d2), and 4(d2)). No significant difference was observed between the treatment groups and control group.

On day 0 of the 1% silver sulfadiazine group, no difference was observed in fibroblast proliferation, collagen formation, vascularization, epithelisation, and inflammatory cell infiltration, and the epidermis layer was observed as damaged due to the burn injury (Figures 2(a3), 3(a3), and 4(a3)). On the 7th day of the 1% silver sulfadiazine group, a severe inflammatory cell infiltration was observed, and in some subjects a slight increase in fibroblast proliferation, vascularization, and epithelisation occurred (Figures 2(b3), 3(b3), and 4(b3)). On the 14th day of the 1% silver sulfadiazine group, a decrease was observed in inflammatory cell infiltration and the fibroblast proliferation, vascularization, and collagen formation were obvious. The epithelisation was detected at medium degree (Figures 2(c3), 3(c3), and 4(c3)). On the 21st day of the 1% silver sulfadiazine group, a decrease in vascularization and inflammatory cell infiltration was observed, and severe fibroblast proliferation and collagen formation were observed (Figures 2(d3), 3(d3), and 4(d3)). No significant difference was observed between the treatment groups and control group.

On day 0 of the 0.9% sodium chloride group, no changes were observed in fibroblast proliferation, collagen formation, vascularization, epithelisation, and inflammatory cell infiltration, and the epidermis layer was observed as damaged due to the burn injury (Figures 2(a4), 3(a4), and 4(a4)). On the 7th day of the 0.9% sodium chloride group, a severe inflammatory cell infiltration was observed and in some subjects there were slight increases in fibroblast proliferation, vascularization, and epithelisation (Figures 2(b4), 3(b4), and 4(b4)). On the 14th day of the 0.9% sodium chloride group, there was a decrease in the inflammatory cell increase, and the fibroblast proliferation, vascularization, and collagen formation were obvious. The epithelisation was detected at medium degree (Figures 2(c4), 3(c4), and 4(c4)). On the 21st day of the 0.9% sodium chloride group, a decrease was observed in vascularization and inflammatory cell infiltration, and there were severe epithelisation, fibroblast proliferation, and collagen formation (Figures 2(d4), 3(d4), and 4(d4)). No significant difference was observed between the treatment groups and control group.

## 4. Discussion

Different surface agents are used in burn injury treatments. The basic purpose is to speed the epithelial healing up and to choose the methods that will prevent the formation of a scar in a wise manner [5]. The method in topical burn injury treatments depends on the depth of the injury and on the treatment targets [6].

While growth hormones and cytokines considerably support the healing of burn wound, suppressor hormones affect the healing of burn wound negatively [7–11]. Therefore, growth hormones, cytokines, and also pharmacological agents that influence receptors of target tissue positively are used for effective treatment of wound healing [12].

Maghsoudi et al. [13] have suggested the use of silver in wound healing, since it has antimicrobial effects on wound infections; silver has negative effects on wound healing though.

Povidone-iodine plays an indirect role in wound healing through controlling the infection. But it is disputable in the cases where iodine is absorbed excessively, which may cause systemic complications. Use of iodine is suggested only in the cases where iodine absorption is limited [14, 15].

Khorasani et al. [16] conducted a study in which they formed an experimental burn injury and showed that the use of saffron gives better results when compared with the use of silver sulfadiazine.

In the study conducted by Eski et al., they performed experimental burn injuries and compared the use of cerium nitrate and saline. They showed that the systemic increase in neutrophil, indicating that the inflammation did not change in the group receiving saline and decreased in the group receiving cerium nitrate [17].

In the study by Sezer et al., they performed experimental burn injuries and performed the assessment of the use of fucoidan-containing pharmaceutical agents in burn injuries treatment. They examined the fibroblast proliferation, inflammatory cell infiltration, epithelisation, and collagen increase in the burn injuries which were similar to those of our study on the 7th, 14th, and 21st days of their experiments. They showed that the inflammatory cell increase was severe on the 7th day and that the fibroblast and collagen increase was at maximum levels on the 14th and 21st days [18].

In our study we compared the effects of the sulfadiazine cream, antiseptic solution povidone-iodine, and 0.9% NaCl serum physiologic on the recovery process of the burn injuries. This comparison was not performed before. In the study we performed second degree burns and determined that there was inflammatory cell infiltration on the 7th day; vascularization, fibroblast proliferation, and collagen increase on the 14th day; and fibroblast proliferation on the 21st day. We also determined that there were no statistically significant differences between the groups in which the collagen increase was the highest.

No statistically significant differences were determined between the healing effects of the agents used in treatment groups in this study. The finding that there are no differences might be related with the depth and/or width of the burn injury or there might not be any differences between the treatment groups in fact.

## 5. Conclusion

In the current study, although these agents have different mechanisms of action, it has been determined that there were no significant differences between the effects of the silver sulfadiazine, povidone-iodine, and sodium chloride 0.9% on healing process of 2nd degree burns. The determination of this effect according to the model created in this study does not mean that the same effect will occur in different burn injury models, and it might be deduced that generally there will not be a clear difference in 2nd degree burns.

## Figures and Tables

**Figure 3:**
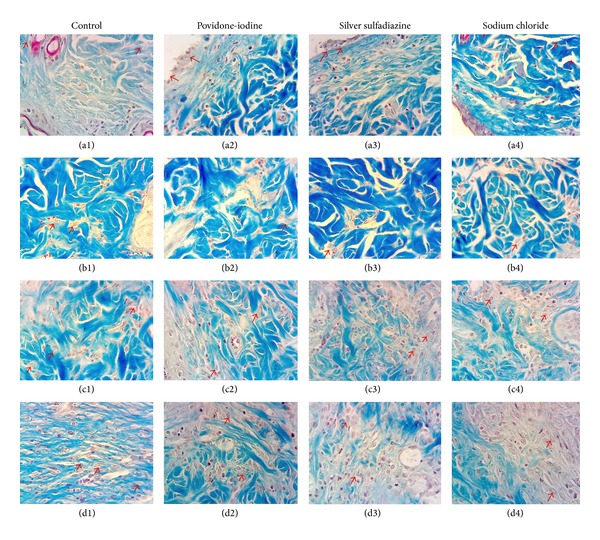
Day 0 ((a1), (a2), (a3), and (a4)), day 7 ((b1), (b2), (b3), and (b4)), day 14 ((c1), (c2), (c3), and (c4)), and day 21 ((d1), (d2), (d3), and (d4)) of Masson trichrome staining. The arrows in (a1), (a2), (a3), and (a4) (→) show epidermis damage. The arrows in (b1), (b2), (b3), and (b4) show the fibroblasts which are few in number. The arrows in (c1), (c2), (c3), and (c4) (→) show a clear fibroblast increase and collagen formation. The arrows in (d1), (d2), (d3), and (d4) show a severe fibroblast increase and collagen formation. (×400).

**Figure 4:**
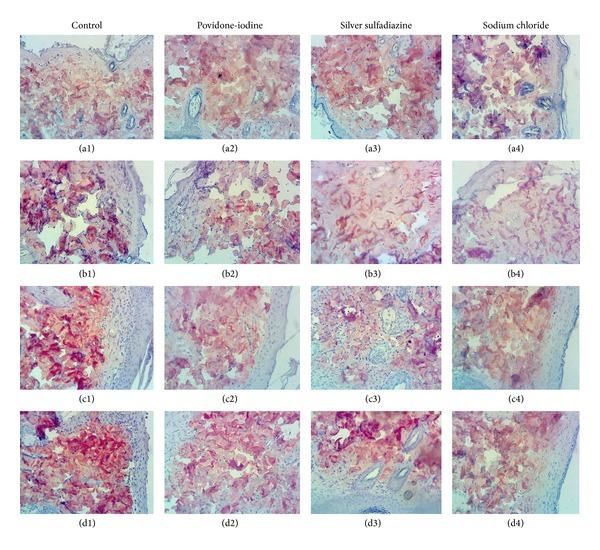
Day 0 ((a1), (a2), (a3), and (a4)), day 7 ((b1), (b2), (b3), and (b4)), day 14 ((c1), (c2), (c3), and (c4)), and day 21 ((d1), (d2), (d3), and (d4)) of type I collagen immune-reactivity (×200).
